# The Regulatory Role of Histone Modification on Gene Expression in the Early Stage of Myocardial Infarction

**DOI:** 10.3389/fcvm.2020.594325

**Published:** 2020-11-30

**Authors:** Jinyu Wang, Bowen Lin, Yanping Zhang, Le Ni, Lingjie Hu, Jian Yang, Liang Xu, Dan Shi, Yi-Han Chen

**Affiliations:** ^1^Department of Physiology, Shanxi Medical University, Taiyuan, China; ^2^Department of Cardiology, Shanghai East Hospital, Tongji University School of Medicine, Shanghai, China; ^3^Key Laboratory of Arrhythmias of the Ministry of Education of China, Shanghai East Hospital, Tongji University School of Medicine, Shanghai, China; ^4^Department of Cardiology, Ruijin Hospital, Shanghai Jiaotong University School of Medicine, Shanghai, China; ^5^Department of Pathology and Pathophysiology, Tongji University School of Medicine, Shanghai, China

**Keywords:** transcriptome, histone modification, epigenomics, transcription factors, super-enhancer

## Abstract

Myocardial infarction (MI) is a fatal heart disease with high morbidity and mortality. Various studies have demonstrated that a series of relatively specific biological events occur within 24 h of MI. However, the roles of histone modifications in this pathological process are still poorly understood. To investigate the regulation of histone modifications on gene expression in early MI, we performed RNA sequencing (RNA-seq) and chromatin immunoprecipitation sequencing (ChIP-seq) on myocardial tissues 24 h after the onset of MI. The genome-wide profiles of five histone marks (H3K27ac, H3K9ac, H3K4me3, H3K9me3, and H3K27me3) were explored through ChIP-seq. RNA-seq identified 1,032 differentially expressed genes (DEGs) between the MI and sham groups. ChIP-seq analysis found that 195 upregulated DEGs were modified by change of at least one of the three active histone marks (H3K27ac, H3K9ac, and H3K4me3), and the biological processes and pathways analysis showed that these DEGs were significantly enriched in cardiomyocyte differentiation and development, inflammation, angiogenesis, and metabolism. In the transcriptional regulatory network, *Ets1, Etv1*, and *Etv2* were predicted to be involved in gene expression regulation. In addition, by integrating super-enhancers (SEs) with RNA-seq data, 76 DEGs were associated with H3K27ac-enriched SEs in the MI group, and the functions of these SE-associated DEGs were mainly related to angiogenesis. Our results suggest that histone modifications may play important roles in the regulation of gene expression in the early stage of MI, and the early angiogenesis response may be initiated by SEs.

## Introduction

Myocardial infarction (MI) is a leading cause of death all over the world, mainly caused by acute coronary artery occlusion, which leads to severe myocardial ischemia, and subsequent myocardium necrosis (1). A considerable number of biological events occur in the early stage of MI, such as inflammation, apoptosis, autophagy, and angiogenesis, leading to ventricular remodeling (2–4). Studies have demonstrated that myocardial pathological changes are mostly reversible, and intervention is more effective during this period (5, 6). Thus, it is of great significance to explore the key molecular events and regulatory mechanisms in the early stage of MI.

Histone modification is a post-translational modification including methylation, acetylation, phosphorylation, ubiquitylation, and SUMOylation, etc. (7), among which the methylation and acetylation of H3K4, H3K9, and H3K27 were common histone marks. Generally, acetylation correlates with transcriptional activation, such as acetylation of histone H3 on lys9 and lys27 (H3K9ac and H3K27ac) (8). Unlike acetylation, the transcriptional activity of methylation modifications on lysine residues varies according to the degree and site of methylation. Tri-methylation of histone H3 on lys9 and lys27 (H3K9me3 and H3K27me3) represents transcriptional suppression, whereas tri-methylation on lys4 (H3K4me3) is associated with transcriptional activation (9, 10).

Super-enhancers (SEs) are characterized as clusters of enhancers, which are enriched with transcription factors, cofactors, and epigenetic modifications, and considered to contain high transcription activity (11). Some enhancer-associated histone modification marks such as H3K27ac can be used to identify SEs (12). Emerging evidences suggest that SEs play important roles in differentiation, development, and tumorigenesis (13–15).

Previous studies had uncovered gene expression changes at the transcription level after MI. The expression of inflammatory response-related genes were reported to be significantly upregulated after MI, which resulted from the activation of NF-κB and TNF signaling pathways (16, 17). However, little is known about the roles of histone modifications and SEs in the early stage of MI. Here, by combining transcriptome and ChIP-seq of key histone modifiers, we set to reveal epigenetic patterns of differentially expressed genes and their roles in the early stage of MI.

## Materials and Methods

### Mouse MI Model

The animal experiments were approved by the Ethics Committee of Tongji University. The mouse model of MI was established by permanent ligation of the left anterior descending (LAD) coronary artery as described previously (18). Briefly, 8-week male C57BL/6 mice were subjected to LAD coronary artery ligation under anesthesia. Sham-operated mice of the same age underwent the same procedure without LAD occlusion. Twenty-four hours after operation, the infarct border zones were dissected, snap-frozen in liquid nitrogen, and stored at −80°C for subsequent experiments.

### RNA Isolation

Myocardial tissues from the infarct border zones of MI and sham group were collected for RNA isolation. Five biological replicates were used for each group. Briefly, total RNA was extracted with 1 ml of RNAiso Plus reagent (Takara, 9108). After adding 200 μl of chloroform, the sample was mixed thoroughly and incubated at room temperature for 3 min, then centrifuged at 12,000 g for 15 min at 4°C. The supernatant was transferred to a new Eppendorf tube on ice, mixed with an equal volume of ice-cooled isopropanol, and then centrifuged at 12,000 g for 15 min at 4°C after placing on ice for 10 min. The supernatant was discarded, and the pellet was washed with 75% ice-cooled ethanol three times, and RNA was dissolved in DEPC water.

### Chromatin Immunoprecipitation (ChIP) Assay

ChIP was carried out with the SimpleChIP® Plus Sonication Chromatin IP kit (Cell Signaling Technology, 56383) according to the manufacturer's protocol. In brief, three biological replicates of myocardial tissues for each group from the infarct border zones were cross-linked with 1% formaldehyde for 10 min, then terminated by adding glycine. After disaggregating the samples into suspensions using Dounce homogenizer, the chromatins were isolated and sonicated to 100–300 bp fragments. Immunoprecipitation (IP) was performed with antibodies against H3K9me3 (Abcam, ab8898), H3K9ac (Active Motif, 39137), H3K4me3 (Abcam, ab8580), H3K27me3 (Cell Signaling Technology, 9733S), and H3K27ac (Active Motif, 39133). Finally, the chromatins were eluted, and then cross-links were reversed. The immunoprecipitated DNA and DNA input were purified using spin columns.

### RNA Sequencing (RNA-Seq) and Analysis

The concentration and quality of RNA were measured by Qubit 4 Fluorometer (Invitrogen™, Q33238). For each group, two biological replicates of isolated RNA above (1 μg total RNA) with RIN > 8 were used for mRNA library construction. RNA sequencing libraries were generated with KAPA mRNA Hyper Prep kits (Roche, KK8581). The libraries were sequenced on an Illumina NovaSeq 6000 platform using a 2^*^ 150-bp sequencing kit for double-ended sequencing.

The raw data were first evaluated by FastQC (v0.11.3). Trimmomatic (v0.36) (19) was applied to remove adaptors, and information of clean reads in RNA-seq data was listed in Supplementary Table 1. Clean reads were then mapped to the mouse genome (mm10) using Hisat2 (20), and the mapping rates of clean reads are listed at Supplementary Table 2. The sequencing alignment data were evaluated by Qualimap (21), and the coverage of gene bodies is shown in Supplementary Figure 1. The counts of genes in each sample were calculated using HTSeq (v0.9.1) (22) and normalized to fragments per kilobase million (FPKM) for further visualization. The raw counts of genes were used to identify differentially expressed genes (DEGs) between sham and MI groups through DESeq2 R package (v1.36.0) (23) with a false discovery rate (FDR) P_FDR_ value < 0.05 and |Log_2_ (Fold change)| ≥ 1. Gene Ontology (GO) and Kyoto Encyclopedia of Genes and Genomes (KEGG) pathway analyses were conducted by R package clusterProfiler (v3.12.0) (24).

### ChIP Sequencing (ChIP-Seq) and Analysis

VAHTSTM Universal DNA Library Prep Kit for Illumina® V3 kit (Vazyme, ND607-02) was used to construct the ChIP-DNA library. Three biological replicates for MI group and two biological replicates for sham group were used for ChIP-seq library construction. The libraries were sequenced on an Illumina NovaSeq 6000 platform. Clean ChIP-seq reads were mapped to the mouse genome (mm10) using bowtie2 (25). To identify the H3K4me3, H3K9me3, H3K9ac, H3K27me3, and H3K27ac peaks, peaks of enriched occupancy relative to a background input were called using MACS 1.4 (26). The peaks were then annotated by the R package ChIPseeker (27) (v1.22.1). Peaks of each histone modification were merged by bedtools (v2.29.2) (28); unique peaks for each group were identified by MAnorm (v1.2.0) (29) software and confirmed by Diffbind R package (v2.14.0) (30). Diffbind R package was used to do the clustering. Hypergeometric optimization of motif enrichment (HOMER) (31) was used to discover motifs of specific regions. The bam files were converted into bigwig files using bedtools (v2.29.2) (28), and then bigwig files were visualized through deeptools (v3.3.0) (32) to generate metagene plots.

### Quantitative Real-Time Quantitative Polymerase Chain Reaction (qRT-PCR) and ChIP-qPCR

Total RNA was reversely transcribed into cDNA with the PrimeScript RT reagent kit (Takara, RR036Q, Japan). qRT-PCR assays were performed in five biological replicates. The reaction volume of real-time qPCR is 20 μl, containing 10 μl of SYBR Green Master Mix (ABI, 4309155, USA), 4 μl cDNA, and 1 μl forward and reverse primers, the primers are listed in Supplementary Table 3. The relative gene expression was calculated with the 2^−ΔΔCt^ method. ChIP-qPCR was performed with 20 μl of reaction volume, containing 10 μl of SYBR Green Master Mix, 1 μl of forward and reverse primers, 2 μl of DNA, and 6 μl of H_2_O. ChIP DNA enrichment was calculated as % of input, and the primers are listed in Supplementary Table 3. All qPCR assays were performed in three biological replicates.

### Identification of Typical and Super-Enhancers

Ranking of super-enhancer (ROSE) (11) was applied to call typical enhancers (TEs) and super-enhancers (SEs). In brief, H3K27ac peaks with the distance shorter than 12.5 kb were merged and ranked by the H3K27ac signal. The inflection point with the tangent line slope equals 1 was the dividing point of TEs and SEs. Enhancers above or below the point were defined as SEs and TEs, respectively. The genes that were closest to the enhancers were considered as enhancer-associated genes. For the expression of enhancer-associated genes, the Wilcoxon rank sum test was performed to test statistical significance between two groups.

## Results

### Transcriptomic Analysis and Histone Modification Profiling

**Figure 1:**
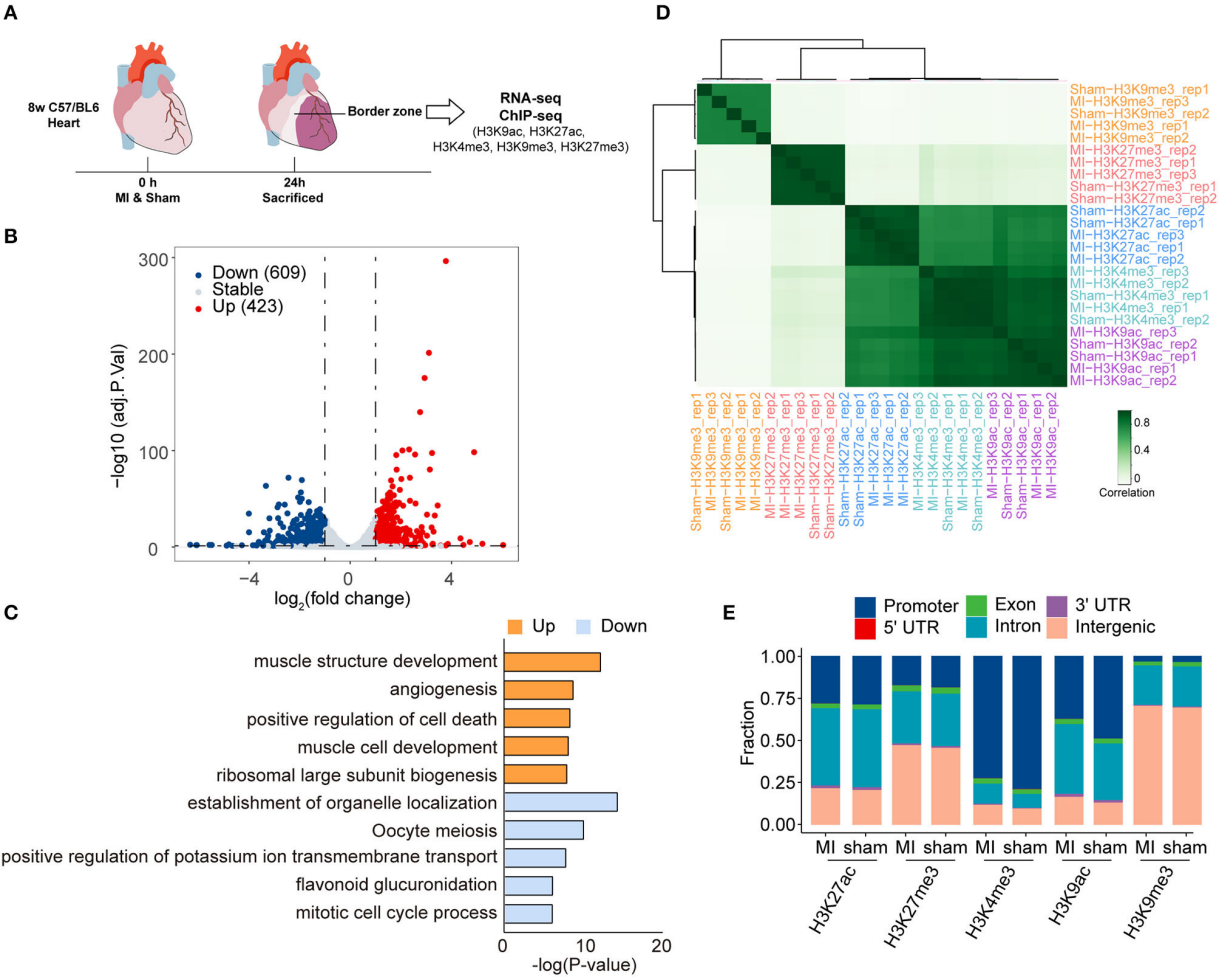
Transcriptome analysis and histone modification profiling in the early stage of myocardial infraction (MI), **(A)** Schematic illustration of the present study. **(B)** Volcano plots showing the differentially expressed genes (DEGs) between sham and MI groups. Blue dots represent downregulated genes, and red dots represent upregulated genes. **(C)** Gene ontology (GO) analysis of the differentially expressed genes (DEGs) in RNA-seq. Orange bras represent the enrichment of upregulated genes; blue bars represent the enrichment of downregulated genes. **(D)** Hierarchical clustering of H3K9ac, H3K9me3, H3K27ac, H3K27me3, and H3K4me3 occupancy at genome wide in sham and MI group. Spearman correlation coefficient was used to calculate sample correlations. **(E)** The genomic distribution of different histone modification regions in sham and MI groups.

Adult C57/BL6 mice (8 weeks old) were subjected to ligation of left anterior descending (LAD) or sham surgery randomly. The mice were sacrificed 24 h after the surgery, and the left ventricular tissues in the infarct border zone and normal tissues in the corresponding position were dissected to examine the expression profile of MI and sham groups by RNA-seq. The quality of the clean reads and mapping rates of RNA-seq are shown in Supplementary Tables 1, 2, and Supplementary Figure 1. Differentially expressed genes (DEGs), 1,032, between the MI and sham groups were identified by RNA-seq with an absolute log-fold change over or equal to 1 (adjusted *P*-value < 0.05) (Figure 1B, Supplementary Table 4). Among them, 423 (40.99%) and 609 (59.01%) genes were upregulated and downregulated, respectively, in the MI group. DEGs, 428 (41.47%), in our RNA-seq data, were overlapped with the DEGs from cardiac organoid model of human myocardial infarction (GSE113871, Supplementary Figure 2). Moreover, the expression of representative cardiac remodeling-related genes (*Nppa* and *Nppb*) was significantly increased in the MI group (Supplementary Figure 3A), which demonstrated the credibility of our MI models. We further performed qRT-PCR to validate the mRNA expression of *Nppa, Nppb*, and *Myh7* in samples both 24 and 48 h after MI. The expression of *Nppa* and *Nppb* increased in 24 h after MI, and then decreased in 48 h after MI (Supplementary Figure 3B). The upregulated genes were enriched in the function of muscle structure development, angiogenesis, positive regulation of cell death, muscle cell development, and ribosomal large subunit biogenesis. In contrast, downregulated genes were enriched in the establishment of organelle localization, oocyte meiosis, positive regulation of potassium ion transmembrane transport, flavonoid glucuronidation, and mitotic cell cycle process (Figure 1C).

To profile epigenetic changes in the early stage of MI, we selected H3K27ac, H3K9ac, and H3K4me3 (three marks associated with active regulatory regions) and H3K9me3 and H3K27me3 (two marks associated with repressive regulatory regions) antibodies to perform ChIP-Seq (Figure 1A). Hierarchical clustering demonstrated that these histone marks were mainly divided into three clusters (Figure 1D). While H3K9me3 and H3K27me3 (two marks associated with repressive regulatory regions) formed two separate clusters, interestingly, all the marks associated with active regulatory regions (H3K27ac, H3K9ac, and H3K4me3) formed a big cluster, suggesting that the active regions might be modified by multiple active histone marks. We then quantified peaks of different histone marks in genomic regions, the number of which was similar between MI and sham groups (Figure 1D). The numbers of peaks of different histone marks and the statistical information of sequencing reads are shown in Table 1 and Supplementary Figure 4. The distribution of genomic regions modified by H3K27ac, H3K27me3, H3K4me3, H3K9ac, and H3K9me3 was classified into six regions (promoter, exon, 3′ UTR, 5′ UTR, intron, and intergenic). The H3K4me3 and H3K9ac marks were mainly mapped to promoters; H3K27ac and H3K27me3 marks were mapped to promoters, intron, and intergenic regions, and H3K9me3 marks were mostly mapped to intergenic regions (Figure 1E), which were consistent with the reported distribution modes of these histone marks (33).

**Table 1:** Information of the peaks called in different samples.

**ID**	**Clean reads**	**Mapped reads**	**Mapped rate (%)**	**Number of peaks**	**Total length of peaks**	**Average length of peaks**
MI24-input_rep1	59,329,700	57,982,915	97.73			
MI24-input_rep2	48,793,596	47,954,346	98.28			
MI24-input_rep3	73,326,196	72,145,644	98.39			
MI24-H3K27ac_rep1	55,638,462	52,016,398	93.49	46,521	153,946,563	3,309.18
MI24-H3K27ac_rep2	48,204,386	47,539,165	98.62	43,004	146,496,195	3,406.57
MI24-H3K27ac_rep3	58,656,804	57,536,459	98.09	50,192	156,520,708	3,118.44
MI24-H3K27me3_rep1	63,728,706	62,568,843	98.18	31,551	112,026,446	3,550.65
MI24-H3K27me3_rep2	51,994,578	51,282,252	98.63	26,864	106,249,613	3,955.09
MI24-H3K27me3_rep3	57,951,114	56,896,403	98.18	38,652	143,281,067	3,706.95
MI24-H3K4me3_rep1	61,403,310	60,193,664	98.03	17,023	41,311,218	2,426.79
MI24-H3K4me3_rep2	46,460,220	45,763,316	98.5	16,072	38,907,043	2,420.8
MI24-H3K4me3_rep3	65,778,746	64,318,457	97.78	20,150	50,138,955	2,488.29
MI24-H3K9ac_rep1	51,708,148	50,761,888	98.17	24,819	64,439,753	2,596.39
MI24-H3K9ac_rep2	37,375,860	35,981,740	96.27	32,902	78,887,608	2,397.65
MI24-H3K9ac_rep3	58,058,296	57,077,110	98.31	42,726	122,678,660	2,871.29
MI24-H3K9me3_rep1	58,645,664	57,437,563	97.94	19,934	36,535,935	1,832.85
MI24-H3K9me3_rep2	46,441,950	45,568,841	98.12	19,821	34,529,866	1,742.08
MI24-H3K9me3_rep3	58,184,316	56,671,523	97.4	23,662	41,583,430	1,757.39
Sham-input_rep1	65,704,850	64,574,726	98.28			
Sham-input_rep2	54,637,390	53,675,771	98.24			
Sham-H3K27ac_rep1	58,613,224	57,739,886	98.51	49,891	160,041,629	3,207.83
Sham-H3K27ac_rep2	51,299,442	50,545,340	98.53	44,392	125,831,890	2,834.56
Sham-H3K27me3_rep1	59,835,338	58,429,207	97.65	35,099	136,040,218	3,875.9
Sham-H3K27me3_rep2	51,733,232	50,734,780	98.07	36,054	113,291,599	3,142.28
Sham-H3K4me3_rep1	48,939,496	48,229,873	98.55	16,772	37,584,277	2,240.89
Sham-H3K4me3_rep2	58,911,408	57,462,187	97.54	17,218	42,505,603	2,468.67
Sham-H3K9ac_rep1	53,257,144	52,421,006	98.43	29,625	84,340,323	2,846.93
Sham-H3K9ac_rep2	56,017,696	55,059,793	98.29	26,963	74,877,575	2,777.05
Sham-H3K9me3_rep1	52,185,442	51,167,825	98.05	21,950	35,551,960	1,619.68
Sham-H3K9me3_rep2	52,790,998	51,756,294	98.04	22,301	37,809,809	1,695.43

### Epigenetic Profile Changes

**Figure 2:**
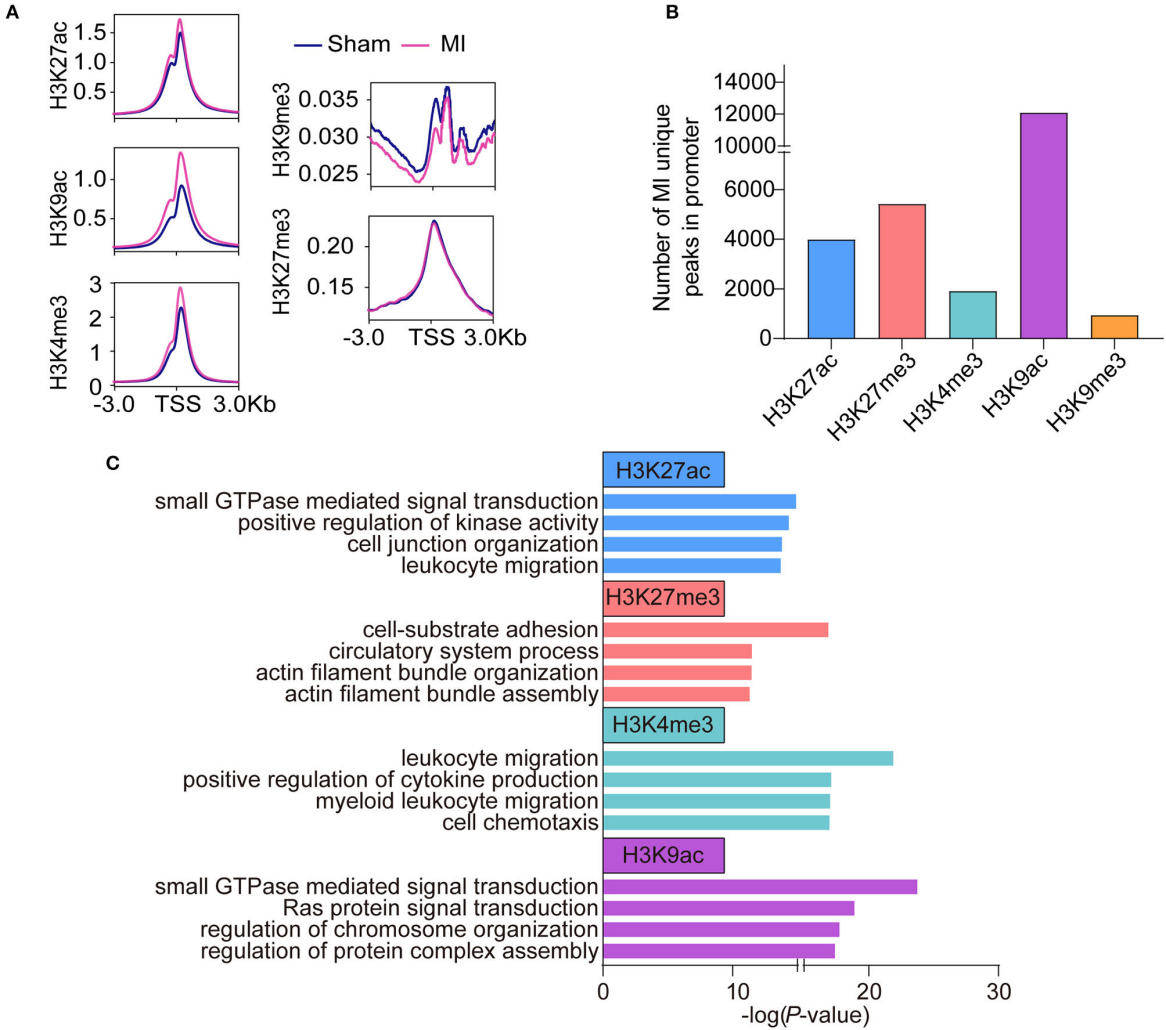
Epigenetic profile change in the early stage of MI. **(A)** The average signals of each histone mark relative to ±3 kb of the transcription start site (TSS) between the sham and MI group. **(B)** The number of MI unique peaks in promoters of H3K9ac, H3K9me3, H3K27ac, H3K27me3, and H3K4me3 marks. **(C)** GO analysis of the MI unique peaks in promoters of H3K9ac, H3K27ac, H3K27me3, and, H3K4me3 marks.

To reveal the difference in histone modifications between MI and sham, we drew the average signals of histone marks relative to ±3 kb of the transcription start site (TSS) (Figure 2A). The average signals of active histone marks (H3K27ac, H3K4me3, and H3K9ac) were all higher in MI than those in the sham group. The signals of H3K9me3 in MI group were relatively lower than those in the sham group, while no apparent changes in H3K27me3 were observed between sham and MI. We identified 3,986 MI unique peaks for H3K27ac mark, 5,417 peaks for H3K27me3 mark, 1,910 peaks for H3K4me3 mark, 12,084 peaks for H3K9ac mark, and 937 peaks for H3K9me3 mark in the TSS ± 3 kb (Figure 2B and Supplementary Tables 5–9). We then performed gene ontology (GO) analysis to investigate the biological function of these MI unique peaks of each histone mark and discovered that the MI unique peaks of H3K27ac (2,778 mapped genes) were highly enriched in small GTPase-mediated signal transduction, H3K27me3 (3,325 mapped genes) in cell-substrate adhesion, H3K4me3 (1,500 mapped genes) in the process of inflammatory response, and H3K9ac (6,547 mapped genes) in small GTPase mediated signal transduction (Figure 2C).

### Active Chromatin Regions Associated With the Upregulated Genes

**Figure 3:**
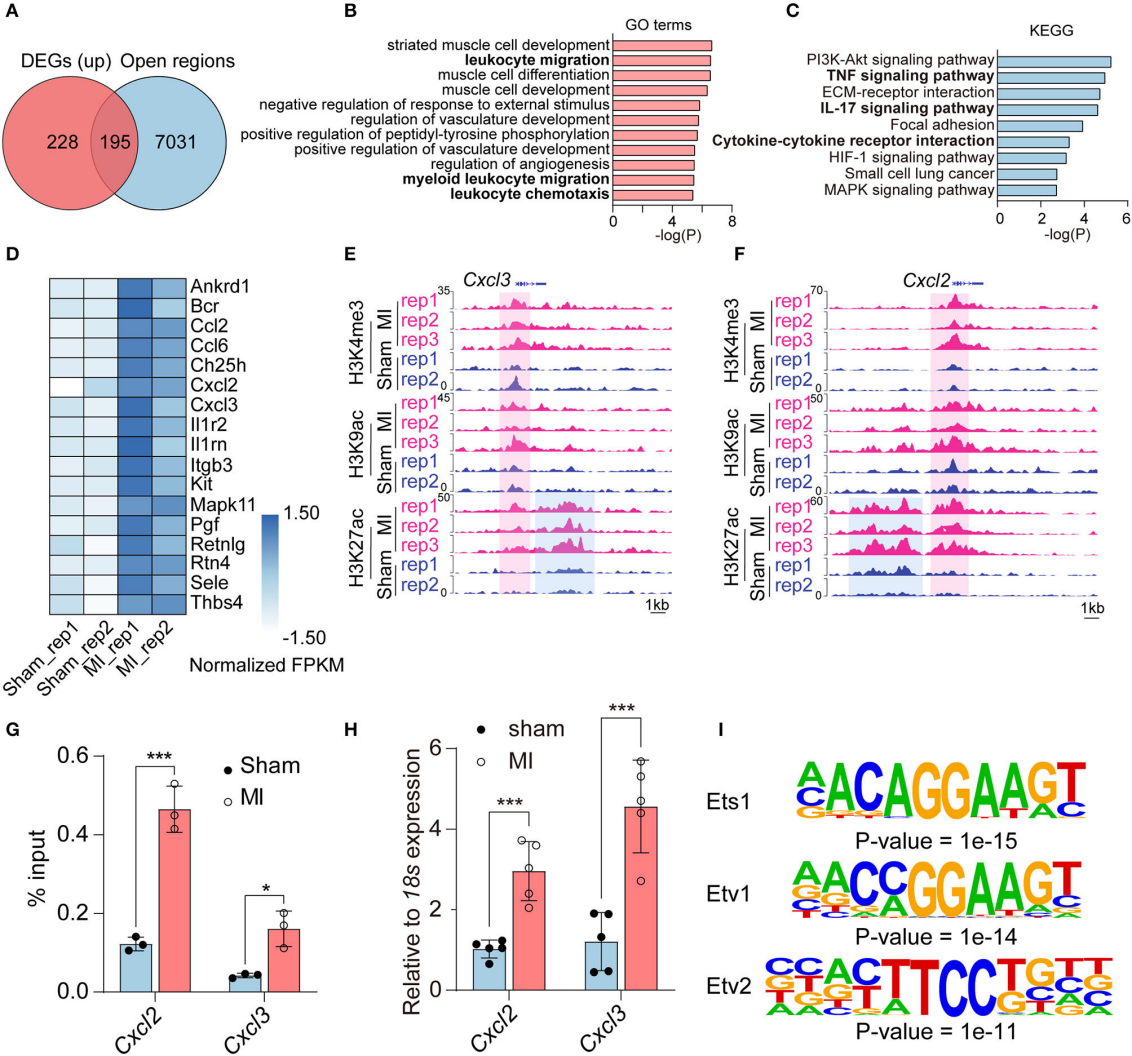
The upregulated genes associated with gain of active histone marks. **(A)** Venn diagram showing upregulated DEGs associated with the open regions in the promoters (gain of H3K9ac, H3K27ac, and H3K4me3 marks in the ±2 kb from TSS). **(B)** GO analysis of the upregulated DEGs enriched in promoters (±2 kb from TSS) of H3K9ac, H3K27ac, and H3K4me3 marks. **(C)** Kyoto Encyclopedia of Genes and Genomes (KEGGs) pathway enrichment analysis of the upregulated DEGs enriched in promoters (±2 kb from TSS) of H3K9ac, H3K27ac, and H3K4me3 marks. **(D)** Heatmap showing the relative expression fragments per kilobase million (FPKM) of genes related to inflammatory response processes. **(E,F)** The increased active histone marks at promoters and enhancers of *Cxcl2* and *Cxcl3* in MI compared with the sham group. **(G)** ChIP-qPCR validation of the distribution of H3K27ac changes in *Cxcl2* and *Cxcl3*. **(H)** qPCR validation of the expression of *Cxcl2* and *Cxcl3*. **(I)** The transcription factors and binding motifs identified at promoter regions of upregulated DEGs enriched for H3K9ac, H3K27ac, and H3K4me3. *n* = 3; *P < 0.05, ***P < 0.001.

Notably, there were few overlapped genes between downregulated genes in RNA-seq and unique peaks in repressive histone marks (Supplementary Figure 5B). Thus, we mainly study the regulation of histone modification on upregulated genes; we intersected the 423 activated genes in RNA-seq [Log2 (fold change) ≥ 1, adjusted *P*-value < 0.05] with 7,226 open regions (the gain of active marks H3K9ac, H3K27ac, and H3K4me3 in the ±2 kb from TSS). A total of 195 activated genes were enriched for at least one of H3K9ac (145/195), H3K27ac (79/195), or H3K4me3 (59/195) marks (Figure 3A, Supplementary Figure 6, and Supplementary Table 10). According to our pipeline, we further validated the 195 activated genes revealed by another method (Diffbind), which also identifies differential peaks. Of the genes, 42.05% (82/195) were shared by the two methods (Supplementary Figure 5A and Supplementary Table 11). We also checked the other 113 genes, which were not identified by Diffbind in genome browser. Most of the promoter regions of the 113 genes did have more active modifications in MI group than that in sham group. We then performed GO analysis to categorize the biological function of the 195 upregulated DEGs and found that they were mainly enriched in muscle cell-related processes (striated muscle cell development, muscle cell differentiation, muscle cell development), inflammatory response processes (leukocyte migration, negative regulation of response to external stimulus, myeloid leukocyte migration, leukocyte chemotaxis) and angiogenesis-related processes (regulation of vasculature development, positive regulation of vasculature development, and regulation of angiogenesis) (Figure 3B). KEGG analysis indicated that inflammatory-related signaling pathways, including TNF signaling pathway, IL-17 signaling pathway, and cytokine–cytokine receptor interaction; cell signal-related pathways (PI3K-Akt signaling pathway and MAPK signal pathway); ECM–receptor interaction and HIF-1 signaling pathways were activated in the process of MI (Figure 3C). The inflammatory response processes and pathways were revealed by enrichment analysis, and inflammatory response-related genes are shown in Figure 3D. Two typical genes (*Cxcl2* and *Cxcl3*) are shown in Figures 3E,F, with increasing H3K4me3, H3K9ac, and H3K27ac signals at the promoter or increasing H3K27ac signals at the enhancer region of *Cxcl2* and *Cxcl3* loci in MI group. ChIP-qPCR and RT-qPCR verified elevated H3K27ac modification in promoter regions and elevated expression of *Cxcl2* and *Cxcl3*, respectively (Figures 3G,H).

Moreover, to identify the transcription factors, which may regulate the upregulated genes in MI, we performed motif analysis at the promoter regions of unique peaks of three active marks (H3K27ac, H3K9ac, and H3K4me3) in the MI group. Hypergeometric optimization of motif enrichment (HOMER) was employed to identify top enriched motifs, which showed that motifs for transcription factors including ETS1 (*P*-value = 1e-15), ETV1 (*P*-value = 1e-14), and ETV2 (P-value = 1e-11) were significantly enriched in promoters of upregulated genes associated with active histone marks (Figure 3I and Supplementary Table 12).

### Identification of MI-Specific SEs

**Figure 4:**
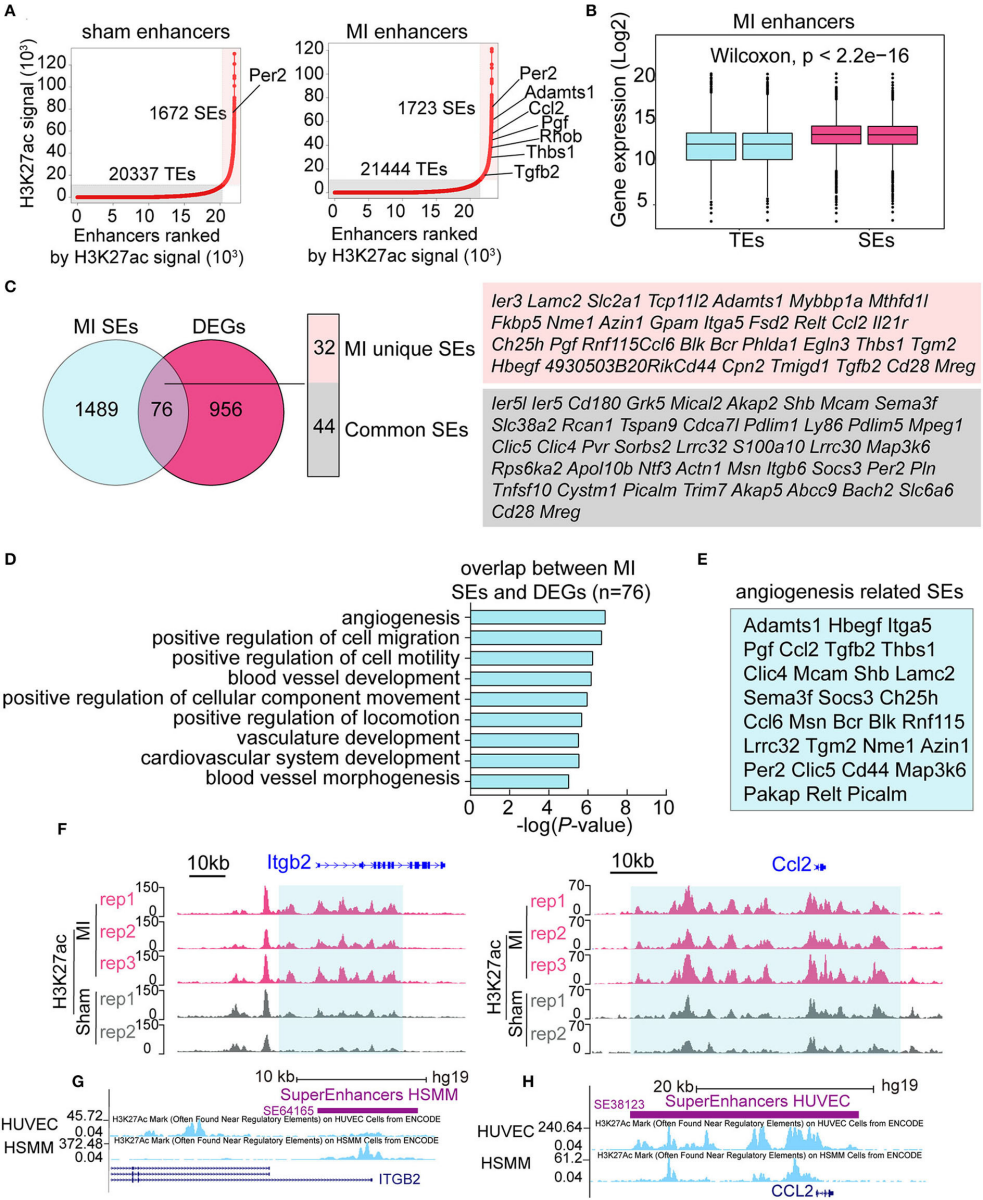
Identification of MI specific super-enhancers (SEs). **(A)** Ranked plots of typical enhancers (TEs) and super enhancers (SEs) in the sham and MI group by increasing H3K27ac signal (normalized by the ratio to the highest H3K27ac reads of the enhancer). The axes values represent the ranks of enhancers by H3K27ac signal. Angiogenesis-related genes are marked out in the figures. **(B)** The expression (Log_2_) of TE-associated genes vs. SE-associated genes in the MI group. *P*-value was determined by the Wilcoxon test. **(C)** Overlapping genes of DEGs and SE-associated genes in the MI group. The MI unique SE-associated genes and common SE-associated genes are listed. **(D)** GO analysis of the 76 overlapping genes between DEGs and SE-associated genes in the MI group. **(E)** The specific genes in the angiogenesis-related GO terms. **(F)** The H3K27ac occupancy of two representative angiogenesis-related genes *Itgb2* and *Ccl2* is shown. The red and gray peaks, respectively, mean MI and sham group. **(G,H)** *Itgb2*- and *Ccl2*-associated SEs on human umbilical vein endothelial cells (HUVECs) and human skeletal muscle myoblasts (HSMM) from ENCODE project.

The above data showed that the signal of H3K27ac was notably higher in the early stage of MI (Figure 2A). H3K27ac is also an active TE and SE marker; however, little is known about the function of SEs in the early stage of MI. We identified 20,337 TEs and 1,672 SEs in the sham group, and 21,444 TEs and 1,723 SEs in the MI group (Figure 4A), showing more enhancers activated in the MI group. Many SE-associated genes, such as *Per2, Adamts1, Ccl2, Pgf* , *Thbs1*, and *Tgfb2*, were known to be related to angiogenesis. Expression levels of SE-associated transcripts in MI were significantly higher than those of TE-associated transcripts (Figure 4B). SEs, 1,723, in the MI group were mapped to 1,565 SE-associated genes, and 76 SEs in the MI group were in accordance with DEGs, which can be further categorized into 32 MI unique SEs and 44 common SEs (Figure 4C). We then performed GO analysis for the 76 SEs associated DEGs and found that about half of the differentially expressed SE-associated genes were enriched in the function of angiogenesis (Figure 4D), and the specific genes in the angiogenesis-related GO terms are listed in Figure 4E. In addition, SEs that were not connected with DEGs were mainly enriched in actin filament-based process (actin filament organization, muscle organ development, regulation of actin filament-based process, lamellipodium organization, and assembly) and cell migration (Supplementary Figure 7). Two representative MI SE-associated genes *Itgb2* and *Ccl2* are shown in Figure 4F, whose H3K27ac occupancy of some component enhancers in the MI group was higher than that in the sham group. *Itgb2*- *and Ccl2*-associated SEs can also be validated on the human umbilical vein endothelial cells (HUVEC) and human skeletal muscle myoblasts (HSMM) from ENCODE database (Figures 4G,H). Our analysis identified a set of SEs, which might be essential for the early response of MI and suggested that MI-induced global changes in SEs might coordinate with angiogenesis.

## Discussion

In this study, we correlated histone modifications to gene expression in the early stage of MI via integrative analysis of the transcriptome and ChIP-seq of histone marks. The biological processes of the upregulated DEGs modified by active marks (H3K9ac, H3K27ac, and H3K4me3) were enriched in cardiomyocyte differentiation and development, inflammation, and angiogenesis. Pathways implicated in the pathological processes of MI were activated, such as inflammation and metabolism-related pathways. Transcription factors, including ETS1, ETV1, and ETV2, may be involved in the transcriptional regulation of molecular events in the early stage of MI. Moreover, a set of SE-associated genes related to angiogenesis were also uncovered.

It is worth noting that inflammatory response was enriched in both function (GO) and pathway (KEGG) analysis, and H3K9ac, H3K27ac, and H3K4me3 regulated the expression of critical chemokines in the early stage of MI. After the occurrence of MI, chemokines were generated in injured myocardium, bound to the endothelial surface and extracellular matrix through glycosaminoglycans, and interacted with receptors on the surface of leukocytes to promote trafficking (34). CC and CXC, two main subtypes of chemokines, were significantly increased in infarcted tissue, which mediated leukocyte migration into the injured myocardium after MI (35, 36). Chemokines played a vital role in the inflammatory response and were associated with cardiac injury, repair, and remodeling following MI (36, 37). Our study showed that the expression of *Ccl2, Ccl6, Cxcl2, Cxcl3*, was upregulated and modified by H3K9ac, H3K27ac, and H3K4me3 marks. *Ccl2* and *Cxcl2* were two widely studied chemokines, which belong to the CC and CXC subtypes. *Ccl2* played an important role in recruiting and activating mononuclear cells, and inhibiting *Ccl2* reduced the infarct size and ameliorated the cardiac remolding after MI (38, 39). Meanwhile, *Cxcl2* mediated the recruitment of neutrophils in the inflammatory response and is involved in the occurrence and development of many cardiovascular diseases, including atherosclerosis, ischemic stroke, and myocardial infarction (40). Studies have demonstrated that highly expressed *Cxcl2* may exacerbate myocardial injury and inhibition of *Cxcl2* reduced neutrophil-mediated tissue injury and infarct size after MI (41–43). Our results revealed that these active histone marks increased at the promoter regions and may promote the expression of these key chemokines after MI. Moreover, biological processes related to cardiomyocyte differentiation and development were also enriched. Among them, *Csrp3, Pdlim5, Sorbs2, Rcan1*, and *Acta1*, which were related to cardiac hypertrophy and remodeling, were upregulated and modified by at least one active histone mark. Altogether, these results indicated that H3K9ac, H3K27ac, and H3K4me3 modifications might participate in cardiac remodeling by regulating the inflammatory response.

In order to get the transcription factors that coordinated the transcriptional regulatory network in the early stage of MI, we performed motif analysis and identified ETS1, ETV1, and ETV2. All three transcription factors were members of the ETS family. ETS1 was associated with fibrotic remodeling of the myocardium (44). ETV1 was involved in atrial remodeling and arrhythmia (45). ETV2 was reported to promote angiogenesis and improve myocardial repair after MI (46). These transcription factors may work with active histone modifications to regulate gene expression in the early stage of MI.

Super enhancers (SEs) were first identified as a large cluster of enhancers with the strong binding of transcription factors that drive the expression of cell identity genes (11). It has been reported that SEs mediate angiotensin II-induced vascular smooth muscle cell dysfunction (47), whereas their roles in MI were less known. In our study, we investigated the function of SEs in MI. We intersected SEs with DEGs and found that 76 DEGs may be regulated by SEs. These SEs can be further categorized into 32 MI unique SEs and 44 common SEs. We then performed GO analysis for the 76 DEGs and found that these genes were significantly enriched in angiogenesis. Angiogenesis after MI is considered to be an important factor leading to ventricular remodeling (48, 49), and neovascularization induced by angiogenic growth factors reduces the infarct size and improves ventricular remodeling (50, 51). Our results showed that SEs might mediate the transcription of angiogenesis-related genes at the border zone in the early stage of MI, consistent with the previous report that angiogenesis initiated in the border zone at the early stage post-MI (52). Moreover, some of these genes have been reported to be associated with angiogenesis in MI, such as *Pgf* and *Ccl2*. *Pgf* (placental growth factor) was reported to promote angiogenesis and myocardial repair post-MI (53, 54). *Ccl2* may be implicated in angiogenesis in ischemic myocardial tissue by attracting macrophages capable of producing angiogenic factors and by exerting direct pro-angiogenesis effects through endothelial cells (36, 55). Apart from SEs, which were associated with DEGs, SEs that were not connected with DEGs were significantly enriched in actin filament-based process and cell migration. These SE-associated genes may remain in a poised status in early MI and participate in the process of cardiac remodeling in the later stage of MI. A representative gene with broad and high levels of H3K27ac signal is *Itgb2*, which mediated the migration and recruitment of endothelial progenitor cells and angiogenesis in the infarcted myocardium (56). Taken together, the transcriptional regulation of angiogenesis-related genes by SEs in the early stage of MI may initiate vascular regeneration and promote repair of damaged myocardium post-MI, which may prevent pathological cardiac remodeling.

Our study also has limitations. We mainly focused on promoters and SEs, while other regulatory elements of gene expression in the early stage of MI were not investigated, which might lead to underestimation of the roles of histone modifications in the early stage of MI. Besides, we only selected five histone marks to study the regulation of histone modification on gene expression in MI; the roles of other histone marks in MI were not investigated. Moreover, the results were based on a mouse model of MI, so further observation and validation in human samples are needed.

In summary, our study revealed the histone modification profile in the early stage of MI in mice and demonstrated that the modulation of histone modifications (H3K9ac, H3K27ac, and H3K4me3) might be involved in inflammation and angiogenesis by regulating promoters and SEs, and participating in the pathological processes of cardiac remodeling.

## Data Availability Statement

All the sequencing results have been uploaded and deposited in the NCBI Sequence Read Archive database (https://www.ncbi.nlm.nih.gov/bioproject/PRJNA657342) under accession No. SRP277594.

## Ethics Statement

The animal study was reviewed and approved by Ethics Committee of Tongji University.

## Author Contributions

Y-HC and DS conceived and coordinated the study. YZ and JW constructed the MI models of mice and collected the samples. JW, YZ, LN, and LH constructed the library for RNA-seq and ChIP-seq and performed some experiments. BL made the bioinformatic analysis of RNA-seq and ChIP-seq and drew the figures. JW drafted the manuscript. JY and LX conducted and gave advice on the study. Y-HC, JY, DS, and BL reviewed and revised the manuscript. All authors have approved the final version of the manuscript.

## Conflict of Interest

The authors declare that the research was conducted in the absence of any commercial or financial relationships that could be construed as a potential conflict of interest.
