# Supplementary material for: The Regulatory Role of Histone Modification on Gene Expression in the Early Stage of Myocardial Infarction
Source: Front Cardiovasc Med. 2020 Nov 30;7:594325. doi: 10.3389/fcvm.2020.594325 (PMC7734124; doi:10.3389/fcvm.2020.594325)
Supplement: Supplementary file 1 [file Table_1.DOCX]

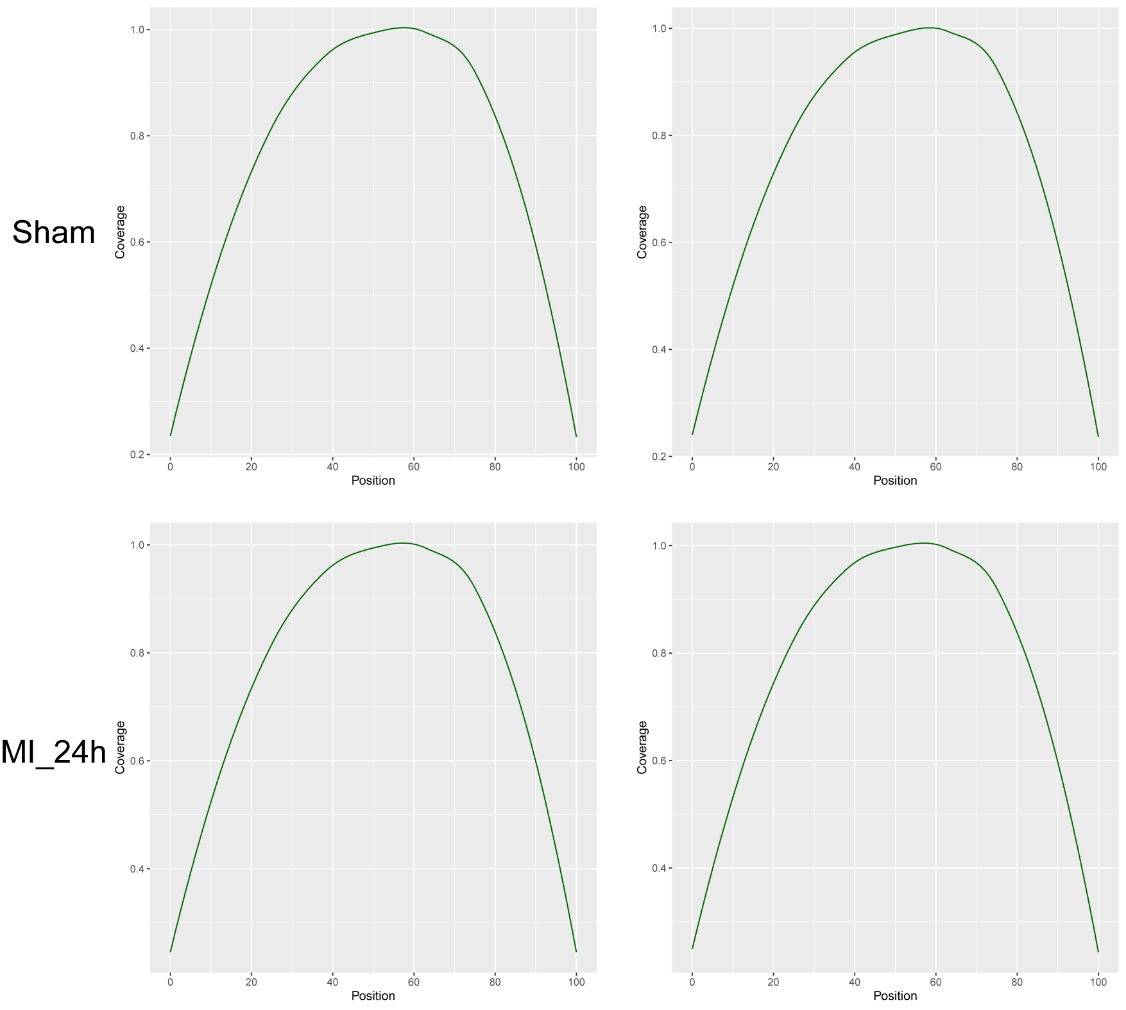


**Supplemental Figure S1.** The coverage of gene bodies of RNA sequencing data in the sham and MI groups.


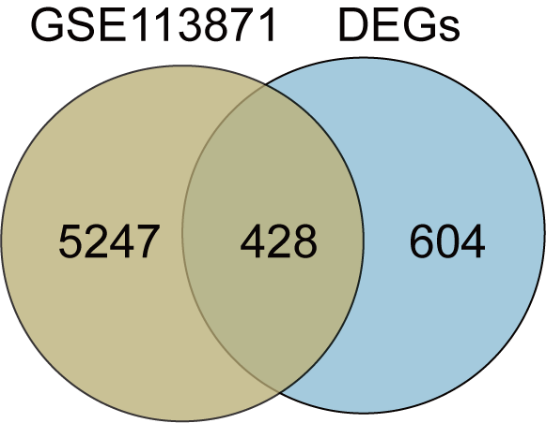


**Supplemental Figure S2.** Overlapping genes of DEGs from cardiac organoid model of human myocardial infarction (P < 0.05) and DEGs in the MI group.


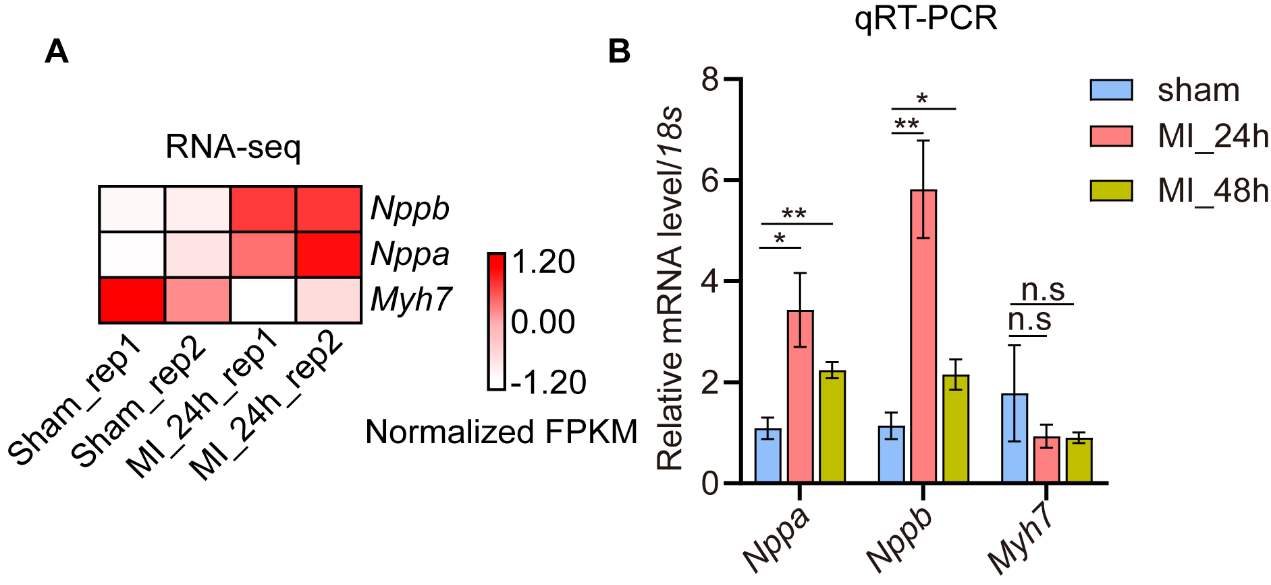


**Supplemental Figure S3. (A)** The expression of cardiac remodeling genes (*Nppb*, *Nppa* and *Myh7*) in RNA-seq. (**B**) The mRNA expression of cardiac remodeling genes (Nppb, Nppa and Myh7) in 24 hours and 48 hours after MI. n = 5; *P < 0.05, **P < 0.01, ***P < 0.001. n.s not significant.


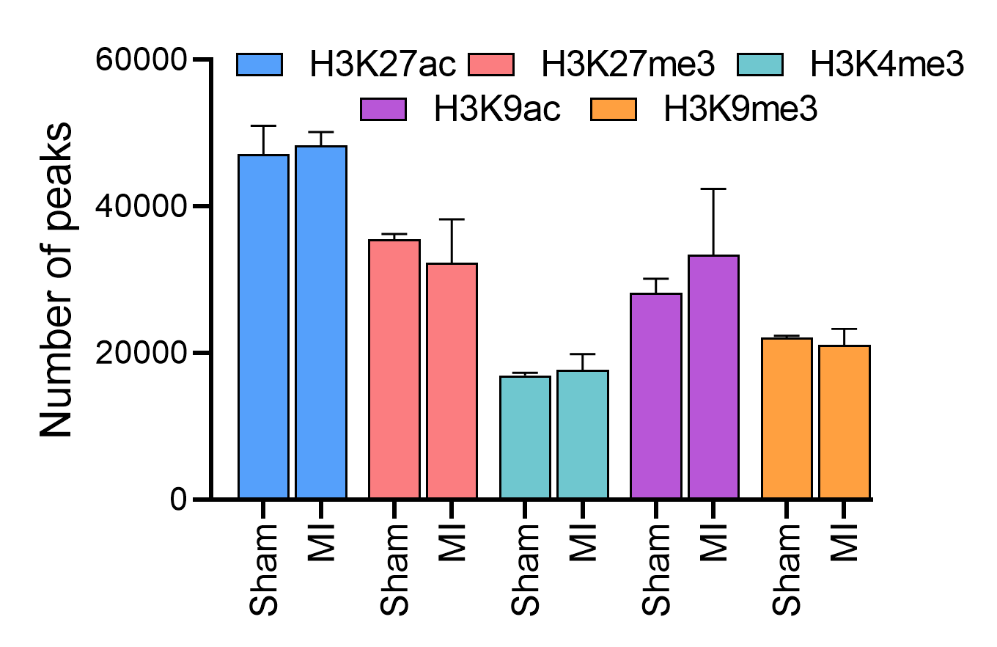


**Supplemental Figure S4.** The number of peaks of different histone marks in the sham and MI groups. n = 2 for Sham group; n=3 for MI group.


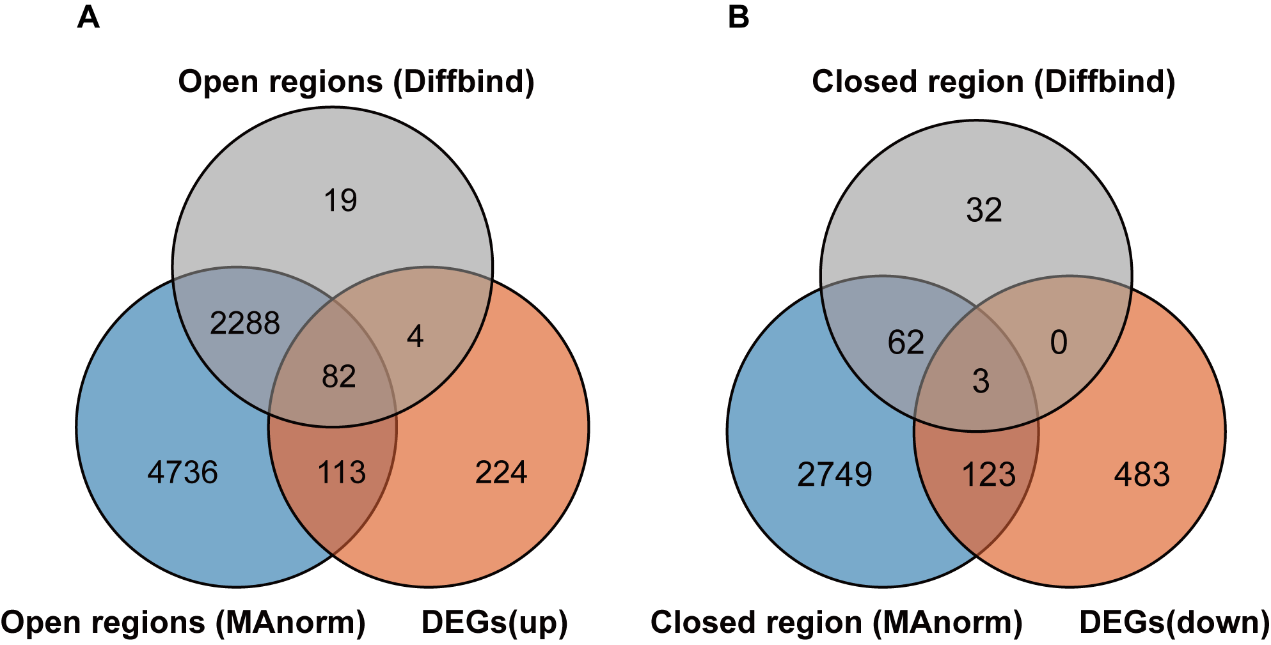


**Supplemental Figure S5. (A**) Venn diagram of the overlapped genes among up-regulated DEGs and open regions analyzed by both DiffBind and MAnorm. (**B**) Venn diagram of the overlapped genes among down-regulated DEGs and closed regions analyzed by both DiffBind and MAnorm.


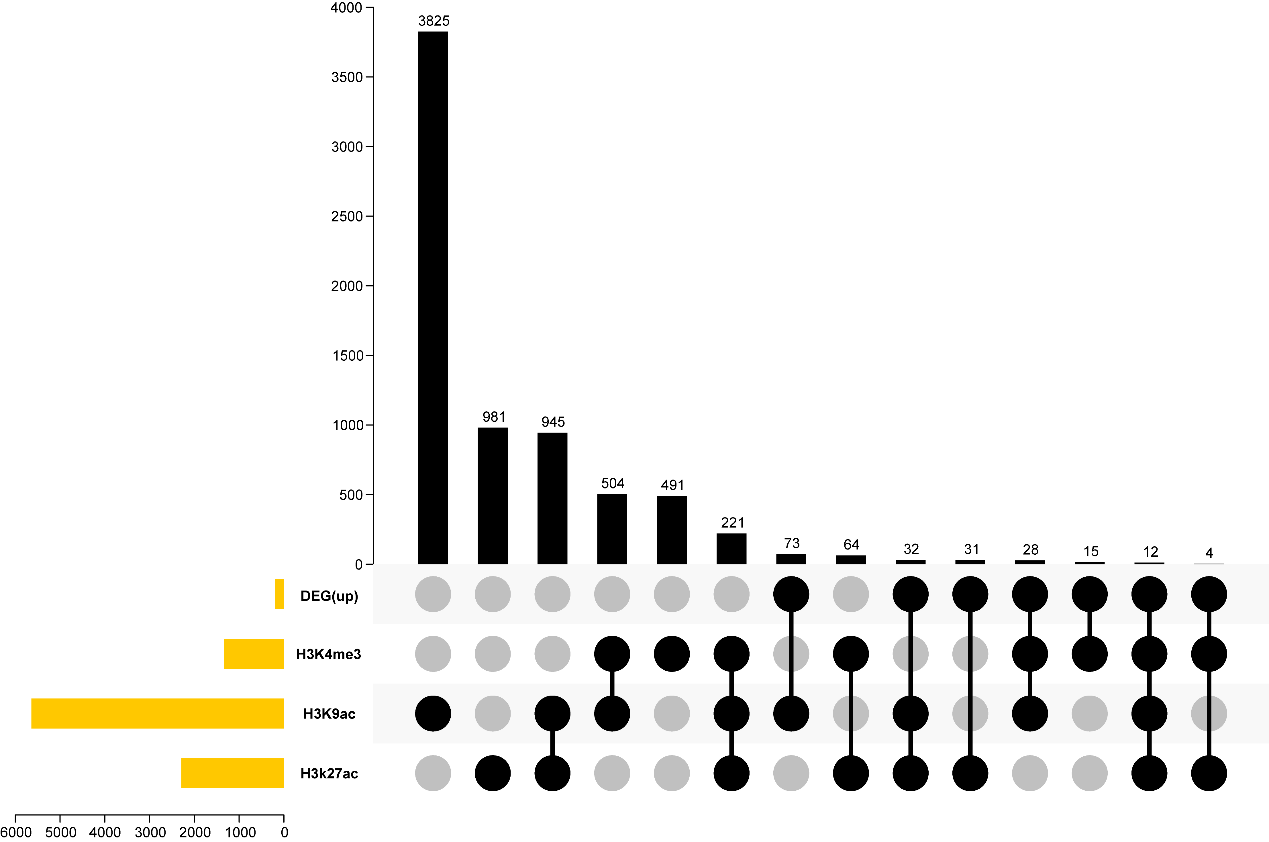


**Supplemental Figure S6.** The UpSet plot of the overlapped genes among the up-regulated DEGs and the gain of H3K9ac, H3K27ac and H3K4me3 marks in promoters.


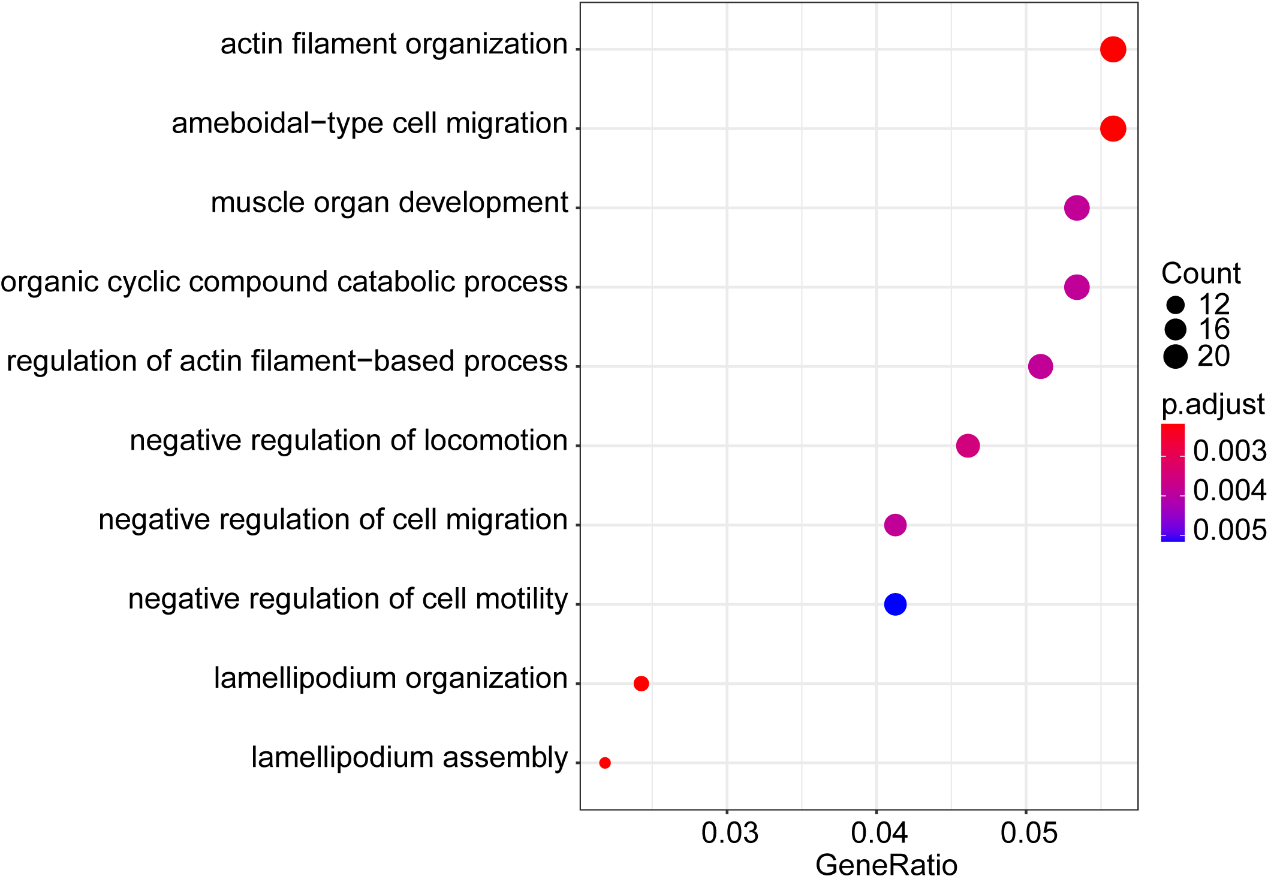


**Supplemental Figure S7.** GO analysis of the MI specific SEs that were not connected with DEGs.
