# Supplementary material for: The Regulatory Role of Histone Modification on Gene Expression in the Early Stage of Myocardial Infarction
Source: Front Cardiovasc Med. 2020 Nov 30;7:594325. doi: 10.3389/fcvm.2020.594325 (PMC7734124; doi:10.3389/fcvm.2020.594325)
Supplement: Supplementary file 11 [file Table_11.DOCX]

**Supplemental Table 1. Information of clean reads in RNA-seq data**

| Sample ID | Total Reads | Total Bases | N Reads% | A% | T% | C% | G% | N% | Error% | Q20% | Q30% | GC% |
| --- | --- | --- | --- | --- | --- | --- | --- | --- | --- | --- | --- | --- |
| Sham_rep1 | 102774594 | 15326567301 | 0.14 | 26.5 | 26.45 | 23.35 | 23.7 | 0 | 0.0235 | 98.81 | 95.46 | 47.05 |
| Sham_rep2 | 82461348 | 12291891324 | 0.14 | 26.52 | 26.47 | 23.31 | 23.71 | 0 | 0.0237 | 98.73 | 95.22 | 47.01 |
| MI_24h_rep1 | 94999240 | 14175550346 | 0.14 | 26.74 | 26.68 | 23.11 | 23.47 | 0 | 0.0235 | 98.83 | 95.51 | 46.58 |
| MI_24h_rep2 | 86629238 | 12925109245 | 0.14 | 26.8 | 26.74 | 23.06 | 23.4 | 0 | 0.0241 | 98.59 | 94.8 | 46.45 |

**Supplemental Table 2. The mapping rates of clean reads of Sham and MI group**

| Sample ID | clean reads | mapped reads | mapped rate (%) |
| --- | --- | --- | --- |
| Sham_rep1 | 102774594 | 98262789 | 95.61 |
| Sham_rep2 | 82461348 | 79608185 | 96.54 |
| MI_24h_rep1 | 94999240 | 93460252 | 98.38 |
| MI_24h_rep2 | 86629238 | 85407765 | 98.59 |

**Supplemental Table 4. qPCR and ChIP-qPCR primers used in the study**

| Target genes | Forward | Reverse |
| --- | --- | --- |
| ms-promoter-*Cxcl2* | ACACGTTTGTTGTGAAGCGG | GCTCTCCTGGGGACAGTCTA |
| ms-promoter-*Cxcl3* | CCCTCTTGCTCCGGTGTTTT | ACCACCAAGGACAGCAAGTG |
| ms-*Cxcl2* | CCACCAACCACCAGGCTAC | GCTTCAGGGTCAAGGCAAAC |
| ms-*Cxcl3* | CCACCAACCACCAGGCTAC | GAGGCAAACTTCTTGACCATCC |
| ms-*ANP* | CTGCTTCGGGGGTAGGATTG | GCTCAAGCAGAATCGACTGC |
| ms-*BNP* | TGGGAGGTCACTCCTATCCT | GGCCATTTCCTCCGACTTT |
| ms-*Myh7* | CGGACCTTGGAAGACCAGAT | GACAGCTCCCCATTCTCTGT |
| ms-*18s* | AAACGGCTACCACATCCAAG | CCTCCAATGGATCCTCGTTA |
